# Supplementary material for: Severe mental illness diagnosis in English general hospitals 2006-2017: A registry linkage study
Source: PLoS Med. 2020 Sep 17;17(9):e1003306. doi: 10.1371/journal.pmed.1003306 (PMC7498001; doi:10.1371/journal.pmed.1003306)
Supplement: S1 Text — (DOCX) [file pmed.1003306.s002.docx]

**Study proposal:** Recognition of severe mental illness in English general hospitals.

**Supervisor:** Andrew Sommerlad ([a.sommerlad@ucl.ac.uk](mailto:a.sommerlad@ucl.ac.uk))

27^th^ January 2019

**Background:**

Severe mental illness (SMI) consists of a range of different conditions including schizophrenia and bipolar disorder. People with SMI are more likely than the general population to experience adverse physical outcomes such as increased smoking, body mass index, and antipsychotic medication use which are linked to diabetes and cardiovascular disease. General hospital admissions are therefore more common, with length of stay being longer and more complex than those without SMI. The ability to provide good quality of care relies on services recognising and accurately diagnosing mental health conditions whilst also incorporating them into physical care. However, we currently have little information about how well mental illness is recognised in non-psychiatric general hospital records.

This study will use routinely collected data from South London and Maudsley NHS Trust’s (SLaM) Clinical Record Interactive Search (CRIS) database to identify people with SMI. We will then use NHS general hospital records to link CRIS data and identify whether mental health conditions are correctly being diagnosed and recorded during admissions since 2006.

**Aims:**

- Establish sensitivity (i.e. how frequently mental health conditions are correctly recorded) of SMI diagnosis during general hospital admission
- Examine time-trend changes since 2006
- Identify clinical and sociodemographic predictors for reduced diagnostic accuracy

**Method:**

We will use SLaM CRIS records to identify participants aged 18 years or older who have been clinically diagnosed with schizophrenia or bipolar as identified by ICD-10 code F20-31. Linked Hospital Episode Statistics (HES) will then be used to identify those who have been admitted to general hospital during the study period (2006-2017), and recorded diagnoses during admissions. Variables of interest include:

- General demographics i.e. age, gender, ethnicity
- Mode of referral to SLaM
- Primary and secondary diagnoses
- Date of diagnosis
- Health of the Nation Outcome Scores (for clinical symptoms)
- Socio-economic status, estimated using patient’s address
- Hospital episode statistics admissions, admission and discharge dates, and diagnostic codes

**Analysis:**

Sensitivity will be the proportion of people with schizophrenia or bipolar disorder recorded in CRIS ‘gold standard’ records who also have the diagnosis recorded in subsequent HES records. We will also look at the number of general hospital admissions and the length of stay for these patients. Chi-squared test will be used to investigate trend in sensitivity over time. Multivariable regression will be used to investigate potential predictors of admission.
