# Supplementary material for: Severe mental illness diagnosis in English general hospitals 2006-2017: A registry linkage study
Source: PLoS Med. 2020 Sep 17;17(9):e1003306. doi: 10.1371/journal.pmed.1003306 (PMC7498001; doi:10.1371/journal.pmed.1003306)
Supplement: S1 Table — ICD-10, International Statistical Classification of Diseases and Related Health Problems, Tenth Revision. (DOCX) [file pmed.1003306.s003.docx]

## S1 Table: Primary diagnosis (ICD-10 code) for emergency hospital admissions of people with severe mental illness

| Diagnosis | Codes | Emergency admissions  (n=45,706) | | Any F code recorded | Sensitivity %  (95% CI) |
| --- | --- | --- | --- | --- | --- |
|  |  | **n** | **%** |  |  |
| Certain infectious and parasitic diseases | A,B | 852 | 1.9 | 645 | 75.7 (72.7, 78.5) |
| Neoplasms | C00-D48 | 774 | 1.7 | 529 | 68.4 (64.9, 71.6) |
| Diseases of the blood and blood-forming organs and certain disorders involving the immune mechanism | D50-89 | 776 | 1.7 | 469 | 60.4 (56.9, 63.9) |
| Endocrine, nutritional and metabolic diseases | E | 1,640 | 3.6 | 1,328 | 81.0 (79.0, 82.9) |
| Mental and behavioural disorders | F | 3,589 | 7.9 | 3,589 | 100 (99.9, 100) |
| Diseases of the nervous system | G | 1,346 | 2.9 | 971 | 72.1 (69.7, 74.5) |
| Diseases of the eye, adnexa, ear and mastoid process | H | 177 | 0.4 | 102 | 57.6 (50.0, 65.0) |
| Diseases of the circulatory system | I | 3,111 | 6.8 | 2,230 | 71.7 (70.1, 73.3) |
| Diseases of the respiratory system | J | 5,168 | 11.3 | 4,093 | 79.2 (78.1, 80.3) |
| Diseases of the digestive system | K | 3,300 | 7.2 | 2,415 | 73.2 (71.6, 74.7) |
| Diseases of the skin and subcutaneous tissue | L | 1,271 | 2.8 | 889 | 69.9 (67.3, 72.5) |
| Diseases of the musculoskeletal system and connective tissue | M | 1,387 | 3.0 | 834 | 60.1 (57.5, 62.7) |
| Diseases of the genitourinary system | N | 2,747 | 6.0 | 1,953 | 71.1 (69.4, 72.8) |
| Pregnancy, childbirth and the puerperium | O | 2,558 | 5.6 | 846 | 33.1 (31.3, 34.9) |
| Certain conditions originating in the perinatal period, congenital malformations, deformations and chromosomal abnormalities | P-Q | 17 | 0.04 | 11 | 64.7 (38.3, 85.8) |
| Symptoms, signs and abnormal clinical and laboratory findings, not elsewhere classified | R and Z and U | 9,420 | 20.6 | 5,592 | 59.4 (58.4, 60.4) |
| Injury | S00-T35, T66-T98, V01-X59, X85-Y98 | 4,042 | 8.8 | 2,722 | 67.3 (65.9, 68.8) |
| Poisoning | T36-65 | 3,531 | 7.7 | 2,815 | 79.7 (78.4, 81.0) |
