# Supplementary material for: Severe mental illness diagnosis in English general hospitals 2006-2017: A registry linkage study
Source: PLoS Med. 2020 Sep 17;17(9):e1003306. doi: 10.1371/journal.pmed.1003306 (PMC7498001; doi:10.1371/journal.pmed.1003306)
Supplement: S2 Table — (DOCX) [file pmed.1003306.s004.docx]

## S2 Table: Clinical characteristics of people with severe mental illness admitted to a general hospital, according to whether mental illness was recorded in their hospital records

| Problem with:  (from Health of Nation Outcome Scale subscales) | All Patients  (n=13,786) | | Psychiatric Diagnosis Recorded (n=10,574) | | No Psychiatric Diagnosis Recorded (n=3,212) | | Significance test |
| --- | --- | --- | --- | --- | --- | --- | --- |
| Agitation | 2,273 | 20.5 | 1,846 | 21.8 | 427 | 16.3 | 0.70 (0.62, 0.79) |
| Self-Injury | 904 | 8.2 | 756 | 8.9 | 148 | 5.7 | 0.61 (0.51, 0.74) |
| Alcohol/Drugs | 1,770 | 16.0 | 1,456 | 17.3 | 314 | 12.1 | 0.66 (0.58, 0.75) |
| Cognition | 2,088 | 18.8 | 1,751 | 20.7 | 337 | 12.9 | 0.57 (0.50, 0.64) |
| Physical Illness | 4,066 | 36.6 | 3,371 | 39.8 | 695 | 26.6 | 0.55 (0.50, 0.60) |
| Hallucinations | 4,069 | 36.8 | 3,203 | 37.9 | 866 | 33.1 | 0.81 (0.74, 0.89) |
| Depressed Mood | 3,194 | 28.8 | 2,433 | 28.7 | 761 | 29.1 | 1.02 (0.93, 1.12) |
| Daily Living | 3,648 | 33.0 | 3,037 | 35.9 | 611 | 23.4 | 0.54 (0.49, 0.60) |
| *Missing^a^* | *2,914* | | *2,272* | | *642* | |  |

**Note:** *^a^*HoNOS domain with most missing data.
