# Supplementary material for: Severe mental illness diagnosis in English general hospitals 2006-2017: A registry linkage study
Source: PLoS Med. 2020 Sep 17;17(9):e1003306. doi: 10.1371/journal.pmed.1003306 (PMC7498001; doi:10.1371/journal.pmed.1003306)
Supplement: S3 Table — (DOCX) [file pmed.1003306.s005.docx]

## S3 Table: Disorder-specific sensitivity of general hospital emergency admission records for people with severe mental illness

| Diagnosis in secondary mental health service record (ICD-10 code) | Number of emergency general hospital admissions | Number of admissions with specific ICD-10 diagnosis recorded | Sensitivity  (95% CI) |
| --- | --- | --- | --- |
| Paranoid schizophrenia (F20) | 26,396 | 11,896 | 45.1 (44.5, 45.7) |
| Schizotypal disorder (F21) | 264 | 9 | 3.4 (1.6, 6.4) |
| Persistent delusional disorder (F22) | 2,890 | 264 | 9.1 (8.1, 10.2) |
| Schizoaffective disorders (F25) | 2,639 | 469 | 17.8 (16.3, 19.3) |
| Other nonorganic psychotic disorder (F28) | 452 | 0 | 0 (0, 0.8) |
| Unspecified nonorganic disorder (F29) | 1,300 | 66 | 5.1 (4.0, 6.4) |
| Manic episode (F30) | 614 | 15 | 2.4 (1.4, 4.0) |
| Bipolar affective disorder (F31) | 11,151 | 4,501 | 40.4 (39.5, 41.3) |

**Abbreviations:** ICD-10 = International Classification of Disease, Tenth Revision
