# Supplementary material for: Severe mental illness diagnosis in English general hospitals 2006-2017: A registry linkage study
Source: PLoS Med. 2020 Sep 17;17(9):e1003306. doi: 10.1371/journal.pmed.1003306 (PMC7498001; doi:10.1371/journal.pmed.1003306)
Supplement: S4 Table — (DOCX) [file pmed.1003306.s006.docx]

## S4 Table: Recording of mental illness in people with severe mental illness admitted to general hospitals: by year of first emergency hospital admission

|  | 2006 | 2007 | 2008 | 2009 | 2010 | 2011 | 2012 | 2013 | 2014 | 2015 | 2016 | 2017 |
| --- | --- | --- | --- | --- | --- | --- | --- | --- | --- | --- | --- | --- |
| Mental illness recorded | 214 | 411 | 401 | 436 | 488 | 523 | 573 | 661 | 617 | 676 | 675 | 135 |
| Mental illness not recorded | 234 | 358 | 375 | 439 | 302 | 308 | 221 | 245 | 287 | 241 | 218 | 44 |
| Sensitivity % (95% CI) | 47.8 (43.1, 52.5) | 53.5 (49.9, 57.0) | 51.7 (48.1, 55.2) | 49.8 (46.5, 53.2) | 61.8 (58.3, 65.2) | 62.9  (59.6, 66.2) | 72.2 (68.9, 75.3) | 73.0 (69.9, 75.8) | 68.3 (65.1, 71.3) | 73.7 (70.7, 76.5) | 75.6 (72.6, 78.4) | 75.4 (68.4, 81.5) |
