# Supplementary material for: Severe mental illness diagnosis in English general hospitals 2006-2017: A registry linkage study
Source: PLoS Med. 2020 Sep 17;17(9):e1003306. doi: 10.1371/journal.pmed.1003306 (PMC7498001; doi:10.1371/journal.pmed.1003306)
Supplement: S5 Table — (DOCX) [file pmed.1003306.s007.docx]

## S5 Table: Association of sociodemographic characteristics with recording of mental illness in general hospital records: multivariable regression without Health of Nation Outcome Scale clinical characteristics

| Characteristics | | Mutually Adjusted Multivariable Analysis (n=12,200) | |
| --- | --- | --- | --- |
|  |  | **Odds Ratio (95% CI)** | **P-Value** |
| Age (per 10-year increment) | | 0.95 (0.92, 0.98) | 0.002 |
| Sex | Female (Reference) | 1 |  |
|  | Male | 0.87 (0.79, 0.95) | 0.002 |
| Ethnicity | White (Reference) | 1 |  |
|  | Asian | 1.14 (0.93, 1.39) | 0.220 |
|  | Black African/Caribbean | **1.43 (1.29, 1.59)** | **< 0.001** |
|  | Mixed | 1.17 (0.88, 1.56) | 0.276 |
|  | Other | **1.24 (1.02, 1.51)** | **0.031** |
| Marital Status | Married (Reference) | 1 |  |
|  | Single | **0.67 (0.59, 0.75)** | **< 0.001** |
|  | Divorced | **0.73 (0.62, 0.87)** | **< 0.001** |
|  | Widowed | **0.71 (0.56, 0.90)** | **0.005** |
| Deprivation Score (per 10-unit increase) | | 1.00 (0.96, 1.05) | 0.843 |

**Note:** Multivariable analysis adjusted for age, sex, ethnicity, marital status, deprivation score, clinical symptoms and function and log number of hospital admissions
