# Supplementary material for: Severe mental illness diagnosis in English general hospitals 2006-2017: A registry linkage study
Source: PLoS Med. 2020 Sep 17;17(9):e1003306. doi: 10.1371/journal.pmed.1003306 (PMC7498001; doi:10.1371/journal.pmed.1003306)
Supplement: S6 Table — (DOCX) [file pmed.1003306.s008.docx]

## S6 Table: Association of clinical and sociodemographic characteristics with psychiatric diagnosis of people with severe mental illness not being recorded in general hospital records: multivariable regression with multiple imputation for missing variables

| Sociodemographic and  Clinical Characteristics | | | Mutually Adjusted Multivariable Analysis (n=13,786) | |
| --- | --- | --- | --- | --- |
|  |  |  | **Odds Ratio (95% CI)** | **P-Value** |
| Age (per 10-year increment) | | | 0.99 (0.96, 1.03) | 0.652 |
| Sex | Female (Reference) | | 1 |  |
|  | Male | | 0.85 (0.78, 0.93) | <0.001 |
| Ethnicity | White (Reference) | | 1 |  |
|  | Asian | | 1.13 (0.93, 1.38) | 0.207 |
|  | Black African/Caribbean | | **1.42 (1.28, 1.57)** | **< 0.001** |
|  | Mixed | | 1.22 (0.92, 1.62) | 0.159 |
|  | Other | | **1.28 (1.06, 1.54)** | **0.010** |
| Marital Status | Married (Reference) | | 1 |  |
|  | Single | | **0.72 (0.63, 0.81)** | **< 0.001** |
|  | Divorced | | **0.79 (0.66, 0.93)** | **0.006** |
|  | Widowed | | **0.78 (0.62, 0.98)** | **0.030** |
| Deprivation Score (per 10-unit increase) | | | 1.01 (0.97, 1.05) | 0.737 |
| Clinical symptoms and function (Health of the Nation Outcome Scale) domains | Mental Health Subscale | No Symptoms (Reference) | 1 |  |
|  |  | 1 Symptom | 0.90 (0.80, 1.01) | 0.062 |
|  |  | 2 Symptoms | **0.74 (0.64, 0.85)** | **< 0.001** |
|  |  | 3+ Symptoms | **0.63 (0.54, 0.74)** | **< 0.001** |
|  | Problem with Physical Illness | | **0.85 (0.76, 0.96)** | **0.008** |
|  | Problem with Daily Living | | **0.70 (0.62, 0.80)** | **< 0.001** |

**Note:** Multivariable analysis adjusted for age, sex, ethnicity, marital status, deprivation score, clinical symptoms and function and log number of hospital admission
