# Supplementary material for: Severe mental illness diagnosis in English general hospitals 2006-2017: A registry linkage study
Source: PLoS Med. 2020 Sep 17;17(9):e1003306. doi: 10.1371/journal.pmed.1003306 (PMC7498001; doi:10.1371/journal.pmed.1003306)
Supplement: Attachment — Submitted filename: Mansour May 2020 Response to reviewers.docx [file pmed.1003306.s009.docx]

## Manuscript reference: PMEDICINE-D-20-00812R1: Severe mental illness diagnosis in English general hospitals 2006-2017: a registry linkage study

Dear editors,

Thank you for your review of our paper. We would like to thank the reviewers for their comprehensive and helpful comments which we believe have strengthened the article.

Below we list editor and reviewers’ comments (in normal type) and our responses to the reviewer (in bold) with amended manuscript text (in italics).

Yours faithfully,

Hassan Mansour (on behalf of all authors)

| Comments | Responses |
| --- | --- |
| *We'd suggest revising the title according to PLOS Medicine's style, this should include the study design in the subtitle (ie, after a colon) - eg here ": registry linkage study" (or similar). | **We have now changed the title as suggested:**  ‘*Severe mental illness diagnosis in English general hospitals 2006-2017: registry linkage study*’ |
| *Please restructure your abstract using the PLOS Medicine headings (Background, Methods and Findings, Conclusions). The writeup for each subsection should be complete sentences (rather than sentence fragments). | **We have changed the abstract headings as requested and have corrected the sentence fragment.** |
| *At this stage, we ask that you include a short, non-technical Author Summary of your research to make findings accessible to a wide audience that includes both scientists and non-scientists. The Author Summary should immediately follow the Abstract in your revised manuscript. This text is subject to editorial change and should be distinct from the scientific abstract. Please see our author guidelines for more information: <https://journals.plos.org/plosmedicine/s/revising-your-manuscript#loc-author-summary> | **We have now included the author summary section.** |
| *We'd suggest ensuring that the study is reported according to the STROBE guideline (for observational studies), and include the completed STROBE checklist as Supporting Information. 1 Please add the following statement, or similar, to the Methods: "This study is reported as per the Strengthening the Reporting of Observational Studies in Epidemiology (STROBE) guideline (SChecklist)." The STROBE guideline can be found here: http://www.equator-network.org/reporting-guidelines/strobe/. When completing the checklist, please use section and paragraph numbers, rather than page numbers. | **This sentence has now been added to P4 Paragraph 3.**  ‘*This study is reported as per the Strengthening the Reporting of Observational Studies in Epidemiology (STROBE) guideline (SChecklist).*’ |
| *Did your study have a prospective protocol or analysis plan? Please state this (either way) early in the Methods section.  a) If a prospective analysis plan (from your funding proposal, IRB or other ethics committee submission, study protocol, or other planning document written before analyzing the data) was used in designing the study, please include the relevant prospectively written document with your revised manuscript as a Supporting Information file to be published alongside your study, and cite it in the Methods section. A legend for this file should be included at the end of your manuscript.   b) If no such document exists, please make sure that the Methods section transparently describes when analyses were planned, and when/why any data-driven changes to analyses took place.   c) In either case, changes in the analysis-- including those made in response to peer review comments-- should be identified as such in the Methods section of the paper, with rationale. | **We have included our brief prospective study protocol as a supporting document, cited it in the text and explained changes to the analysis plan in the text. E.g.**  ‘*In response to peer-reviewer comments, we additionally calculated sensitivity according to 1) the primary diagnosis recorded for each hospital admission (grouped into ICD-10 categories)and 2) the number of previous hospital admissions (0, 1-5, ≥6).*’ P7 paragraph 3  **and**  **‘***To explore the impact of missing data, we conducted two non-prespecified sensitivity analyses*’ P8 paragraph 1 |

| **Reviewer 1** |  |
| --- | --- |
| Thanks for the opportunity to review your manuscript. My role is as a statistical reviewer, and so my comments and queries focus on the data and the analysis in this work (and the presentation of these). This manuscript aims to estimate the sensitivity of diagnosis for serious mental illness during general hospital admissions (i.e. not to specialist mental health units), and whether this varies according to patient characteristics.  I think this is a well-written and presented manuscript. The sensitivity analyses are appropriate and anticipated my initial thoughts about the missing data issues we usually encounter from routinely-collected data. | **We are pleased that you thought this paper was well-written and presented. Thank you very much for your helpful comments which we think have improved the reporting of our research.** |
| The analyses looking at how patient-level factors are associated with missed diagnosis are estimated using logistic regression. The description of the odds ratios, i.e. 'those from Black African/Caribbean backgrounds were 1.38 … times more likely' is incorrect, as an odds ratio will be a biased estimator of a rate ratio where the outcome (missed recording of the mental health diagnosis) is very common (e.g. ~50% overall). Either the analyses should be updated to estimate a rate ratio (i.e. log-binomial model or similar) or the wording should be adjusted when describing the odds ratios throughout the manuscript. | **Thank you for pointing this out. We have now changed the wording of this result in the manuscript.**  ‘*In mutually adjusted multivariable analysis, those from Black African/Caribbean backgrounds were more likely (odds ratio (OR) = 1.38 (95% CI 1.24, 1.55; p < 0.001)) to have no psychiatric diagnosis ever recorded compared to those from White ethnic backgrounds.’* P9 paragraph 5 |
| I found it slightly confusing to go from the outcome being sensitivity in the first part of the manuscript but the regression analyses then invert the outcome to be missed diagnoses. | **We are sorry that this was confusing. We inverted the outcome to be ‘missed diagnosis’ as we believe that this is the main outcome of interest. We have endeavoured to use clear language in describing these results. Rather than inverting all results in the manuscript, we have instead amended the title of the analysis and results sub-sections to aid clarity and hope that this is satisfactory:**  *‘Association of sociodemographic and clinical factors with psychiatric diagnosis being unrecorded’* P9 subheading 2 |
| P4, Paragraph 1. There are details of the source database and the linkage method used, is there any information available on the quality assurance procedures and the estimate false positive rate of record matching? | **Thank you for this query. We have added to the manuscript to clarify that the linkage was made using deterministic matching procedures**.  ‘*SLaM data were linked using deterministic matching procedures to NHS Digital’s Hospital Episode Statistics data’* P5 paragraph 1  **In a submitted but not yet published paper, our colleagues have examined potential for bias arising from this process and found that 94% were successfully matched and there was little change in risk factor associations after accounting for missed matching bias. Unfortunately, we are unable to cite these unpublished results at present but hope that this information clarifies the utility of this data for the reviewer.** |
| P6, Paragraph 2. Is the 'chi square test for trend' the Cochrane-Armitage test? | **Thank you for this query. We had originally used a Pearson Chi squared test, but having reviewed this analytic approach, we consider that the Cochran-Armitage test is preferable due to the ordinal nature of the categorisation by year. We therefore now provide the result from the Cochran-Armitage test. The main result is unchanged, although the chi-squared statistic is different.** |
| P6, Paragraph 3. What was the fraction of missing information found after imputation, and is the number of imputed datasets appropriate for the observed FMI? | **The fraction of missing information was 0.33 and 20 imputed datasets is therefore likely to be appropriate to give replicable estimates of standard error. We have added this information to the manuscript, citing the following reference:**  *‘The fraction of missing information after imputation was 0.33 so 20 imputed datasets is likely to give replicable estimates of standard error.[1]’* P8 paragraph 1  [1] Von Hippel et al, Sociological methods and research. 2018 <https://doi.org/10.1177/0049124117747303> |
| Which version of Stata was used? | **We used Stata 15 and have added this information to the manuscript.** |
| What type of model was used for each of the variables in the MI with chained equations? i.e. for categorical variables was this logistic or discriminant analysis? | **Thank you, we used predictive mean matching for continuous (IMD), and logistic regression for categorical (ethnicity, marital status, HoNOS categories) data and have added this to manuscript.** |
| P7, Paragraph 4. What was the association between number of hospital admissions, and how would this be interpreted from the regression model/Table 4 given it's log-transformed? (also p25 appendix 5) | **We did not intend to analyse the association of number of hospital admissions with diagnostic accuracy but included this a priori as a covariate. The distribution of the data required it to be log-transformed.**  **In response to your comment, we have now calculated patient-level sensitivity according to the number of previous hospital admissions and we think that this now provides an indication of the effect of repeat hospitalisations on diagnostic recording.**  *‘**Patient-level sensitivity was 57.3% (55.8, 58.8) for patients with only one admission, 80.7% (79.7, 81.6) for those with 2-5 admissions, 92.8% (91.5, 93.9) for those with 6-10 admissions, and 96.7 (95.7, 97.6) for those with 11+ admissions.’* P9 paragraph 1 |
| P7, Paragraph 5. There is an overall increase in trend but Figure 2 reveals that most of the change was from 2009 to 2012, rather than a linear increase over time. | **Thank you we have added this comment to the manuscript:**  ‘*much of this change was observed between 2009 and 2012’* P9 paragraph 4 |
| P11, Paragraph 3. Is there a way with the datasets to zoom in specific hospital admissions where an accurate mental health diagnosis would equivocally be needed? This is more of a curious question than something that needs addressing the manuscript. | **Thank you for this suggestion. We agree that this would be interesting so have therefore undertaken a post-hoc analysis whereby we report admission-level sensitivity according to the primary diagnosis recorded for each hospital admission (grouped into ICD-10 categories).**  *‘Admission-level sensitivity according to primary diagnosis varied from 33.1% (31.3, 34.9) for admissions related to pregnancy, childbirth and the puerperium (ICD-10 codes O00-O99) to 79.7% (78.4, 81.0) for poisoning related admissions (T36-65) ; and 81.0% (79.0, 82.9) for endocrine, nutritional and metabolic diseases (E00-E90) (see S1 Table for full results).’* P9 paragraph 3 |
| P22, Appendix 2. One of the few drawbacks to a large dataset like this is that Chi-square tests effectively just signal a large sample size - I would suggest including a measure of effect size (RR/OR) with corresponding CIs as well as a p-value and summary data. | **Thank you, we have replaced the significance tests in appendix 2 with odds ratios and 95% confidence intervals.** |
| P22, Appendix 4. Is the CI for the sensitivity (here and elsewhere in the MS) from a normal-approximation method or something else? | **We have clarified in the manuscript that we used the Pearson-Clopper method for calculating confidence intervals.**  ‘*We calculated 95% confidence intervals for sensitivity using the Clopper-Pearson method.*’ P7 paragraph 4 |

| **Reviewer #2** |  |
| --- | --- |
| Thanks for the opportunity to peer review this well-conducted study. It addresses an important topic - the disparities in mortality and morbidity, largely due to preventable medical conditions, that are experienced by people with severe mental illnesses, and the role of health service integration and diagnostic practice as mechanisms for this health inequality. The study uses electronic medical records to link specialist mental health service users' clinical and sociodemographic profiles with their episodes of care in emergency medical facilities, and specifically focuses on the accuracy (sensitivity) of psychiatric diagnoses that are recorded in emergency medical care. Overall, this study makes good use of available data to provide examination of a key aspect of care: the methods, measures, and approach to analysis are clearly reported, the ability to adjust for the effects of a range of sociodemographic factors is particularly informative (and the findings and implications for minority ethnic groups with SMI are rightly highlighted), as is the exploration of time trends in diagnostic accuracy over the ten year study period. | **Thank you very much for your comments on our manuscript and we are pleased that you thought that this research is important and well-conducted.**  **We are also grateful for your comments which have helped us to improve the manuscript.** |
| My main concern relates to the extent to which this measurable aspect of care - explicit psychiatric diagnosis recorded during the general medical /emergency episode - may be associated with differential clinical outcome. It clearly is an accessible indicator of integrated care. But a little more consideration of the impacts of this being noted or omitted would be helpful. | **Thank you for this comment. We considered whether we could examine if recording of psychiatric diagnosis was associated with an important clinical outcome, but the data we had available would not allow unbiased analyses. For example, we considered examining the association of diagnostic recording with subsequent number of hospital admissions or length of stay but judged that these outcomes would likely be a function of illness severity rather than diagnostic recording practice. We believe that different study design would be required to consider outcome associated with diagnostic recording and have expanded on our discussion of potential future research in this area:**  **‘…***our observational study design meant that we were unable to examine whether diagnostic recording affected clinical outcomes.*’  *‘Future studies should examine whether diagnostic recording affects outcomes such as length of stay or readmission rate, and has any negative effects such as stigma or diagnostic overshadowing of physical illness. This may require more detailed scrutiny of individuals’ case-records and could be analysed as part of future evaluations of liaison/consultation psychiatry services.’* P13 paragraph 2 |
| The authors stress the importance of 'specific [psychiatric] diagnosis for treatment outcomes and continuity of care'.. and note that this may facilitate '..a referral to more specialist mental healthcare service such as liaison psychiatry'.. and that it will enable '..continuity of previous care such as medication, and tailoring of inpatient and discharge plans to account for comorbidity'.  These are important principles of care, but, could some more practical/ concrete inference or example be made? | **We have added other practical suggestions for how the identification of psychiatric illness in hospital may lead to better clinical practice:**  **‘***Other holistic measures to promote the wellbeing of a general hospital inpatient with severe mental illness may also be instituted after identification of the presence of a psychiatric disorder. These may include consultation with family to identify whether psychiatric symptoms are new or pre-existing, discussion with community mental health teams, or provision of a side room offered so that sleep could be prioritised.*’ P11 paragraph 1  **We have also added to our explanation of the potential work required in general hospital settings to promote integrated mental and physical healthcare:**  ‘*Hospital settings should endeavour to improve diagnostic recording, particularly for high-risk groups. This may include training staff in culturally sensitive diagnosis for ethnic minority populations, expansion of mental healthcare input in general hospitals and collaborative working with these liaison psychiatry services, and proactive contact with primary care and mental health services to elicit information about past psychiatric history.’ P14 paragraph 1* |
| Could it be useful to consider the types of presenting condition in the emergency setting to clarify relationships between diagnosis and care? e.g. Appendix 1 - indicates common primary diagnoses to be injury and poisoning, where mental ill-health may be directly related to event or risk.. or endocrine/metabolic disease where anti-psychotic medication may be especially relevant.. or maybe a broader statement that for many of the presenting problems, psychotropic medications may be directly relevant to management decisions such as prescribing. | **Thank you very much for this interesting idea. We have added an additional post-hoc analysis based on your suggestion in which we report on ‘admission-level’ sensitivity according to the ICD-10 categories in appendix 1:**  *‘Admission-level sensitivity according to primary diagnosis varied from 33.1% (31.3, 34.9) for admissions related to pregnancy, childbirth and the puerperium (ICD-10 codes O00-O99) to 79.7% (78.4, 81.0) for poisoning related admissions (T36-65) ; and 81.0% (79.0, 82.9) for endocrine, nutritional and metabolic diseases (E00-E90) (see S1 Table for full results).’* P9 paragraph 3  **The results regarding the relatively high sensitivity for poisoning and metabolic-related admissions support your hypothesis. We have added to the discussion regarding this analysis:**  *‘Sensitivity appeared to vary according to the primary reason for admission so that admissions for poisoning, which may have been precipitated by mental health symptoms, or those for endocrine-and metabolic conditions which may result from psychotropic adverse effects, had higher diagnostic recording. The lower recording for pregnancy and childbirth-related is of concern and warrants further future investigation, though it may reflect coding practice differences.’* P12 paragraph 3 |
| I have some minor suggestions for the introduction section. The initial quantification of SMI prevalence ('..affect[s] around 3% of the population..') uses lifetime prevalence derived from a single, albeit high quality, general population study in Finland; it could be useful to clarify the statement - at least by noting this rather high estimate is for lifetime prevalence, and the authors might consider alternatively noting the point or period estimates available from systematic review/s. | **We have now changed these figures to reflect findings from systematic review and global figures using the following references:**  *‘Severe mental illnesses (SMI), defined as schizophrenia spectrum and bipolar affective disorders, have lifetime prevalence of around 0.5% (1) and 1% (2) respectively…*‘ P4 paragraph 1   1) Simeone et al, BMC Psychiatry. 2015; **15,** 193 2) Merikangas et al, Arch Gen Psychiatry. 2011;68(3):241-251 |
| The authors note limited studies concerning mental disorder recognition in medical settings, but may wish to modify their statement about the paucity of research in this area, noting that Mitchell et al (2012) have a conducted systematic review of alcohol misuse identification (2012), and they might also allude to the larger body of work (some of which they later cite) on psychosis recognition in primary care. | **Thank you, we did not find this paper in our search for studies examining diagnostic accuracy for psychiatric conditions but have now added this to our manuscript in the introduction and discussion:**  *‘A further study examined recording of alcohol disorders in primary and secondary care*. P4 paragraph 2  **and**  ‘*Sensitivity in this study was higher than the figure of 52% reported for recognition of alcohol disorders by hospital staff.*’ P11 paragraph 2  Mitchell et al. The British Journal of Psychiatry, 2012; *201*(2), 93-100. |

| **Reviewer #3** |  |
| --- | --- |
| In the rationale, the authors describe that understanding coding of psychiatric disorders in general hospitals will facilitate our understanding of the healthcare received for physical needs in people with SMI, i.e. from HES can we identify people with SMI who are in hospital for any reason, and how accurate the diagnostic labels are. I agree this is important, as physical health problems appear to be a main driver of the mortality and morbidity gap. | **Thank you for your review and helpful comments. We agree that this is an important issue.** |
| The main problem is that the primary diagnosis in HES relates to the reason for admission. "The primary diagnosis is the main reason the patient is receiving care in hospital, while the secondary diagnoses are relevant co-morbidities and external causes if these have been identified." - this is taken from a HES metadata guide (available here <https://digital.nhs.uk/data-and-information/publications/statistical/hospital-admitted-patient-care-activity/2018-19>)  Accordingly, someone with SMI being admitted for a respiratory condition, for example, would have a primary code from ICD-10 chapter J, and may have a secondary code about their psychiatric diagnosis from chapter F (if it was recorded!). By focusing only on the primary code, you have primarily selected on psychiatry related admissions, and not on admissions with a primary indication for physical health problems. The HONOS data by recording status in Table 1 and Appendix 6 appear to indicate this. Because your rationale is based on physical health problems, I do not think this was your intention. If it was, then please reframe the paper accordingly (i.e. you wanted to look at psychiatric codes for psychiatric problems admitted through emergency admissions in general hospitals).  My main recommendation is to re-analyse the data using the secondary codes in HES, and then consider the effect of the primary versus secondary codes, perhaps through sensitivity analyses. You could further investigate the issues of coding with reason for admission with the use of consultant speciality codes.  Although this means reconstructing and re-analysing the whole dataset, the value of your findings the research community is such that this is well worth doing. If this is not done, then I think there are only very limited information to be gained as your HES analytic sample is overly selected. | **We are sorry that it was unclear, but our analysis had already been conducted and reported in the manner you suggest.**  **We aimed to examine whether pre-existing psychiatric illness was recorded when individuals were admitted to a general hospital for any reason (which appendix A indicates was usually medical or surgical).**  **We therefore derived our outcome data – i.e. whether psychiatric diagnosis was recorded in hospital records – from HES records and the outcome of interest ‘diagnosis being recorded’ included it being recorded as any one of the 20 discharge diagnoses.**  **We hope this is now clear and have amended the manuscript to clarify this, in the aims section:**  *‘We therefore aimed to evaluate the accuracy of SMI recording during English general hospital admissions of a person with pre-existing SMI who is admitted to a general hospital for any health condition…’* P4 paragraph 3  **Also, in the outcomes section:**  ‘*Our outcome of interest was recording of psychiatric illness in any of the 20 recorded primary or secondary diagnoses.*’ P6 paragraph 2  **And the analysis section:**  ‘*We examined sensitivity of psychiatric diagnosis recording at patient-level (proportion of people with SLaM SMI diagnosis who ever had a mental illness recorded in their complete general hospital records as a primary or secondary diagnosis) and emergency admission-level (proportion who have a mental illness recorded in primary or secondary diagnoses during each emergency admission).*’ P7 paragraph 3 |
| Query the terminology of 'gold-standard' used to describe the diagnosis applied in the SLaM data, perhaps 'reference standard' is more appropriate? What is the 'right' label and how it is arrived at (and who decides) in this context is appropriate to be elaborated on, however briefly, and there is a multitude of literature on this. | **Thank you for your suggestion – we agree that this terminology is more appropriate and have now changed the term ‘gold-standard’ to ‘reference-standard’ throughout the paper.**  **We have also added to the discussion about the utility of diagnostic labels, and how this may influence practice in different settings.**  ‘*The lower sensitivity for more specific diagnoses may also reflect uncertainty about diagnostic labels, including whether these are accurate, and used and communicated appropriately; there is the potential for disagreement between clinicians and for different diagnostic practices in different settings.(1)*’ P10 paragraph 3  **We cite the following study:**  *Perkins et al, The Lancet Psychiatry 2018; 5(9), 747-764*  **We also expand on debate about diagnostic classification in psychiatry in the strengths and limitations section, acknowledging questions about their validity.**  ‘*Although there is wider debate about the validity of psychiatric diagnostic constructs, SMI categories are considered stable and persistent over time.’* P12 paragraph 4  **We cite the following texts:**  *Allsopp et al. Psychiatry research. 2019;* ***279****: 15-22.*  *Huda. The Medical Model in Mental Health: An Explanation and Evaluation. 2019* |
| Could the objectives be made more clear in that they relate to the general hospitals, e.g. … association of sociodemographic and clinical factors with [more] accurate psychiatric recording [in general hospital records] | **Thank you, we have changed this as suggested in all three objectives:**   1. *‘Calculate the sensitivity of SMI diagnosis in general hospital records* 2. *Evaluate time-trend changes of SMI diagnosis recording in general hospital records from 2006 to 2017* 3. *Examine association of sociodemographic and clinical factors with accuracy of psychiatric recording in general hospital records’* P4 paragraph 4 |
| How much influence did the hierarchical classification have? How did you operationalise this, for example if someone has 2 diagnoses in SLaM, but you found a diagnosis of bipolar disorder in HES and they were assigned as having schizophrenia in your algorithm, was this counted as missed code? You find that sensitivity for F31 is lower than for F20, how much of this is due to the method? I may be missing something but I don't see why this classification is necessary if some people have more than one SMI, please either justify, or do not apply the hierarchy. It would be helpful if you provided the number with more than one diagnosis code in SLaM. | **In the example you give, this would not count as a missed code in our primary analysis of sensitivity which was the proportion of people with schizophrenia-like disorder or bipolar disorder (F20-31) who had any mental disorder (F00-99) recorded. It would count as a missed code for our secondary analysis of ‘category-specific sensitivity’. We think this secondary analysis is valuable as it allows us to provide sensitivity estimates at a more specific level of diagnostic accuracy. We discuss this point in paragraph 2 of the discussion.**  **We acknowledged that there is diagnostic uncertainty for some patients presenting with affective and psychotic symptoms. We chose to use this hierarchical classification so that we did not double-count patients as, for example, having both bipolar and schizophrenia: we judged that the coexistence of these diagnoses was unlikely clinically and that these two diagnoses appearing on the same patient’s record was more likely to reflect differing or evolving clinical opinion, and the hierarchical model is an established approach to allocate patients to single diagnostic entities. We have expanded on our justification for this:**  ‘*We did this to avoid double-counting patients who may have been diagnosed at different times within the reference-standard database as having a schizophrenia-like disorder and bipolar disorder; we judged that this most likely represented evolving clinical opinion rather than coexistence of the two disorders, which is clinically unlikely.*’ P5 paragraph 3  **We cite the following study:**  *Benbarre et al. European Psychiatry. 2001;16(3), 167-72*  **We are unfortunately unable to calculate the proportion of patients with diagnoses of both bipolar and schizophrenia with the data we currently have available to us because the hierarchy was applied at the time of data extraction.** |
| Further elaboration on HES, for example its function and purpose, who enters the data, how the diagnoses are arrived at, the purpose and structure of diagnostic data contained within it (e.g. primary versus secondary), and any changes to the recording of these data over the studies time period are needed. | **Thank you, we have added information about this to the manuscript ‘outcomes’ section:**  **‘***The HES database is primarily designed to allocate payment to hospitals for the care they provide, and its secondary uses are for research and health service planning.*’ P6 paragraph 2  **We have also moved information about the recording of diagnoses in HES to this section for clarity:**  *‘Diagnoses recorded in HES include those clinically identified by hospital staff during admissions, those derived from pre-existing clinical records from secondary mental health trusts or previous hospital medical records, or those obtained following communication with primary care. Separate EHRs are used in different hospital trusts, meaning that diagnoses are not automatically entered in general hospital records from secondary mental healthcare or primary care records. Some EHRs may automatically populate diagnosis fields with previously recorded chronic conditions, although there is no data available to determine the extent of this practice.*’ P6 paragraph 3  **We do not have specific information about changes in data recording during the study period but our findings of increasing sensitivity over time provide information about this and we discuss this in the context of changing coding practices over time in the discussion.** |
| You could move the rationale behind focusing on emergency admissions from the sensitivity analysis to the main methods section on HES. Please reference the evidence for rationale. | **Thank you for your suggestion, we have added more detail to the methods section when we describe the HES data source:**  *‘emergency, i.e. unplanned admission, or elective, such as admission for renal dialysis, wound dressing, chemotherapy, or elective surgery’* P6 paragraph 2  **We have retained information about the rationale for our decision to focus on emergency only admissions, and have added the following reference for the HES data dictionary which gives more detail about the classification of these admission types. [1].**   1. Digital N. HES Data Dictionary: Admitted Patient Care. 2018. <https://digital.nhs.uk/binaries/content/assets/legacy/pdf/1/c/hes_data_dictionary_-_admitted_patient_care.pdf> (accessed 16.05.2020) |
| How did the covariates of age, sex, ethnicity and marital status get into the SLaM records? E.g. via an admissions interview, or self-reported questionnaire, or from relatives, or a mixture? Are there any indications of its accuracy? | **Basic sociodemographic information for patients registering in mental health services is usually extracted from NHS Digital’s Spine database and additional information may be added from a combination of clinical data from several different sources. We do not know of any studies which have investigated its accuracy but have no reason to suspect that there would be any systematic inaccuracies which might affect our results.** |
| Please note which address was used to generate IMD, e.g. first, last contact in the dataset? It isn't usual to analyse an IMD mean score; usually IMD is categorised into quintiles or deciles (quartiles for very small datasets). | **All covariate information was derived from the time closest to the first hospital admission and this is outlined in the paper. IMD score was also normally distributed (Skewness = -0.1; Kurtosis = 3.0) so we consider that reporting a mean score and standard deviation is appropriate. Other studies have also reported mean IMD scores e.g.**  **Werbeloff et al, Lancet Psychiatry 2017;4(1), 49-56**  **Perera et al, Age and Aging 2016;45(5),668-673** |
| In figure 1, please add the final number of people with SMI with a discharged admission (i.e. analysed number). It is noted that 13,786 people were admitted, but please clarify in the figure that when the 25,941 discharged admissions were discarded that none of your sample were lost? | **Thank you for spotting this we have now adjusted Figure 1 to highlight that 27 patients were removed for having non-complete first hospital admissions.** |
| Page 7 under Sensitivity of general hospital SMI diagnosis: "We found that 10,574 of 13,786 people with SMI *were diagnosed with any psychiatric illness* during their…" I don't think this is what you meant, but the terminology is also used in the discussion, and in the Interpretation section of the Abstract. The distinction of being 'diagnosed' with SMI in hospital versus 'a pre-existing SMI being noted' could be more clear and consistent. | **Thank you for your suggestion – we agree that this wording was ambiguous here and have now changed the language used from ‘diagnosis’ to ‘recording’ in several places and hope that this is now clear. E.g.**  *‘We found that 10,574 of 13,786 people with SMI had any psychiatric illness recorded during their subsequent general hospital admissions…* ’ P9 paragraph 1 |
| They may both be true, in which case this needs commenting on. As such, it becomes important to understand and comment further on *how* existing diagnoses make it into HES related to patient / informant disclosure, or linkage to other systems? Is there any literature to bring in here? How these diagnoses are recorded is central to your analysis, so consider introducing some of what you write across page 10/11 into the introduction. | **From your earlier suggestion, we made changes to the description of the HES data source in the methods section as we judge this is where this is best placed and hope that this is satisfactory. The literature we are able to cite for this is the detailed ‘data dictionaries’ describing the source.** |
| Related to this is the important point you make about a previous admission being a source of information for a current admission. Did you not consider adding this to your analysis plan? Even though you don't have true 'incident' admission, you might be able to pick this up as a signal. If your aim is to help researchers understand SMI recording in HES better, then pointing them to this potential source of under-reporting in more distant versus recent more admissions might be important. | **Thank you for this suggestion. We adjusted our regression analyses for the number of hospital admissions. The number of admissions variable was log-transformed due to the distribution of data, making the coefficient derived difficult to interpret.**  **However, we agree it is useful to know about how sensitivity varies according to the number of previous admissions, so we have undertaken a post-hoc analysis in which we report patient-level sensitivity according to the number of previous admissions; 1 (i.e. index admission after ‘CRIS diagnosis’), 2-5 previous admissions, 6-10 previous admissions and 11+ previous admissions.**  *‘Patient-level sensitivity was 57.3% (55.8, 58.8) for patients with only one admission, 80.7% (79.7, 81.6) for those with 2-5 admissions, 92.8% (91.5, 93.9) for those with 6-10 admissions, and 96.7 (95.7, 97.6) for those with 11+ admissions.’* P9 paragraph 1 |
